# Supplementary material for: A neural-mechanistic hybrid approach improving the predictive power of genome-scale metabolic models
Source: Nat Commun. 2023 Aug 3;14:4669. doi: 10.1038/s41467-023-40380-0 (PMC10400647; doi:10.1038/s41467-023-40380-0)
Supplement: Supplementary file 3 — Reporting Summary [file 41467_2023_40380_MOESM3_ESM.pdf]

Corresponding author(s): Jean-Loup Faulon
Last updated by author(s): Jun 21, 2023

Reporting Summary

Nature Portfolio wishes to improve the reproducibility of the work that we publish. This form provides structure for consistency and transparency in reporting. For further information on Nature Portfolio policies, see our Editorial Policies and the Editorial Policy Checklist.

Statistics

For all statistical analyses, confirm that the following items are present in the figure legend, table legend, main text, or Methods section.

n/a	Confirmed	
☐	☒	The exact sample size (*n*) for each experimental group/condition, given as a discrete number and unit of measurement
☒	☐	A statement on whether measurements were taken from distinct samples or whether the same sample was measured repeatedly
☒	☐	The statistical test(s) used AND whether they are one- or two-sided<br>*Only common tests should be described solely by name; describe more complex techniques in the Methods section.*
☒	☐	A description of all covariates tested
☒	☐	A description of any assumptions or corrections, such as tests of normality and adjustment for multiple comparisons
☐	☒	A full description of the statistical parameters including central tendency (e.g. means) or other basic estimates (e.g. regression coefficient) AND variation (e.g. standard deviation) or associated estimates of uncertainty (e.g. confidence intervals)
☒	☐	For null hypothesis testing, the test statistic (e.g. *F*, *t*, *r*) with confidence intervals, effect sizes, degrees of freedom and *P* value noted<br>*Give P values as exact values whenever suitable.*
☒	☐	For Bayesian analysis, information on the choice of priors and Markov chain Monte Carlo settings
☒	☐	For hierarchical and complex designs, identification of the appropriate level for tests and full reporting of outcomes
☒	☐	Estimates of effect sizes (e.g. Cohen's *d*, Pearson's *r*), indicating how they were calculated

Our web collection on statistics for biologists contains articles on many of the points above.

Software and code

Policy information about availability of computer code

Data collection

The following data collection tools were used for this study:
- pydoe2 (open-source python package, version: 1.3.0)
- BioTek Gen5 (commercial software, version: 3.03)
- cobrapy (open-source python package, version: 0.22.1)

Data analysis	The following data analysis python packages were used for this study:<br>- tensorflow (open-source, version: 2.6.0)<br>- numpy (open-source, version: 1.20.3)<br>- pandas (open-source, version: 1.3.3)<br>- scipy (open-source, version: 1.7.1)<br>- sci-kit learn (open-source, version: 0.24.2)<br>- keras (open-source, version: 2.6.0)<br>- keras-preprocessing (open-source, version: 1.1.2)<br>- matplotlib (open-source, version: 3.4.3)<br>- seaborn (open-source, version: 0.11.2)<br><br>The repository supporting this article can be found at https://zenodo.org/record/8056442 or https://github.com/brsynth/amn_release

For manuscripts utilizing custom algorithms or software that are central to the research but not yet described in published literature, software must be made available to editors and reviewers. We strongly encourage code deposition in a community repository (e.g. GitHub). See the Nature Portfolio guidelines for submitting code & software for further information.

Data

Policy information about availability of data

All manuscripts must include a data availability statement. This statement should provide the following information, where applicable:

- Accession codes, unique identifiers, or web links for publicly available datasets
- A description of any restrictions on data availability
- For clinical datasets or third party data, please ensure that the statement adheres to our policy

Metabolic models used in this study can be found with the following accessions on the BiGG database: e_coli_core, iML1515, iJN1463. Unidirectional versions of these models can be found on our repository, at https://github.com/brsynth/amn_release/tree/main/Dataset_input/. The original dataset from the ASAP database can be found under the accession Mutant Biolog Data I. The original dataset from Nogales et al. can be found as Supporting Information of the study.
The source data underlying all figures presented in the main manuscript and Supplementary Information (including training sets used in Figs. 3-5), are provided with this paper as a downloadable archive. Additional datasets and raw data are available on our Github repository (cf. Code availability), or from the corresponding authors upon reasonable request.

Human research participants

Policy information about studies involving human research participants and Sex and Gender in Research.

Reporting on sex and gender	N/A
Population characteristics	N/A
Recruitment	N/A
Ethics oversight	N/A

Note that full information on the approval of the study protocol must also be provided in the manuscript.

Field-specific reporting

Please select the one below that is the best fit for your research. If you are not sure, read the appropriate sections before making your selection.

☒ Life sciences ☐ Behavioural & social sciences ☐ Ecological, evolutionary & environmental sciences

For a reference copy of the document with all sections, see nature.com/documents/nr-reporting-summary-flat.pdf

Life sciences study design

All studies must disclose on these points even when the disclosure is negative.

Sample size	The sample size of the experimental dataset produced by the authors of this study is 110. It was chosen based on time, resource, and model's performance considerations. The simulated datasets had various sizes depending as well on time, resource, and model's performance considerations.
Data exclusions	No data was excluded for simulated datasets. For the experimental dataset produced by the authors, outliers showing clear statistical or visual deviation from the other replicates were removed, without any pre-established criteria. The number of preserved replicates range from 2 to 8, with an average of 4.6 (± 1.6) replicates per medium composition.<br>For the external dataset stemming from the ASAP database, we removed data in the pre-processing steps as described in the Methods

section, for compatibility issues with the model. For the external dataset stemming from the J. Nogales et al., no data was excluded.

Replication	For the experimental dataset produced by the authors, each growth rate measure was replicated 8 times (all of them were technical replicates, on the same plate, performed a single time). Some replications failed (no growth observed) and were therefore considered outliers. For external datasets, no replication scheme was performed, to our knowledge.
Randomization	We did not assign samples to experimental groups in our study, therefore randomization is not relevant.
Blinding	We controlled the experimental setups ourselves, so blinding was not relevant in this study.

Reporting for specific materials, systems and methods

We require information from authors about some types of materials, experimental systems and methods used in many studies. Here, indicate whether each material, system or method listed is relevant to your study. If you are not sure if a list item applies to your research, read the appropriate section before selecting a response.

Materials & experimental systems

n/a	Involved in the study	
☒	☐	Antibodies
☒	☐	Eukaryotic cell lines
☒	☐	Palaeontology and archaeology
☒	☐	Animals and other organisms
☒	☐	Clinical data
☒	☐	Dual use research of concern

Methods

n/a	Involved in the study	
☒	☐	ChIP-seq
☒	☐	Flow cytometry
☒	☐	MRI-based neuroimaging
